# What do health professionals need to know about young onset dementia? An international Delphi consensus study

**DOI:** 10.1186/s12913-021-07411-2

**Published:** 2022-01-02

**Authors:** Leah Couzner, Sally Day, Brian Draper, Adrienne Withall, Kate E. Laver, Claire Eccleston, Kate-Ellen Elliott, Fran McInerney, Monica Cations

**Affiliations:** 1 College of Education, Social Work and Psychology, Flinders University, Adelaide, South Australia Australia; 2 School of Psychiatry, UNSW Sydney, Sydney, New South Wales Australia; 3 School of Public Health and Community Medicine, UNSW Sydney, Sydney, New South Wales Australia; 4 College of Medicine and Public Health, Flinders University, Adelaide, South Australia Australia; 5 University of Tasmania, Hobart Tasmania, Australia

**Keywords:** Young onset dementia, Health professionals, Delphi study, Knowledge, Consensus

## Abstract

**Background:**

People with young onset dementia (YOD) have unique needs and experiences, requiring care and support that is timely, appropriate and accessible. This relies on health professionals possessing sufficient knowledge about YOD. This study aims to establish a consensus among YOD experts about the information that is essential for health professionals to know about YOD.

**Methods:**

An international Delphi study was conducted using an online survey platform with a panel of experts (*n* = 19) on YOD. In round 1 the panel individually responded to open-ended questions about key facts that are essential for health professionals to understand about YOD. In rounds 2 and 3, the panel individually rated the collated responses in terms of their importance in addition to selected items from the Dementia Knowledge Assessment Scale. The consensus level reached for each statement was calculated using the median, interquartile range and percentage of panel members who rated the statement at the highest level of importance.

**Results:**

The panel of experts were mostly current or retired clinicians (57%, *n* = 16). Their roles included neurologist, psychiatrist and neuropsychiatrist, psychologist, neuropsychologist and geropsychologist, physician, social worker and nurse practitioner. The remaining respondents had backgrounds in academia, advocacy, or other areas such as law, administration, homecare or were unemployed. The panel reached a high to very high consensus on 42 (72%) statements that they considered to be important for health professionals to know when providing care and services to people with YOD and their support persons. Importantly the panel agreed that health professionals should be aware that people with YOD require age-appropriate care programs and accommodation options that take a whole-family approach. In terms of identifying YOD, the panel agreed that it was important for health professionals to know that YOD is aetiologically diverse, distinct from a mental illness, and has a combination of genetic and non-genetic contributing factors. The panel highlighted the importance of health professionals understanding the need for specialised, multidisciplinary services both in terms of diagnosing YOD and in providing ongoing support. The panel also agreed that health professionals be aware of the importance of psychosocial support and non-pharmacological interventions to manage neuropsychiatric symptoms.

**Conclusions:**

The expert panel identified information that they deem essential for health professionals to know about YOD. There was agreement across all thematic categories, indicating the importance of broad professional knowledge related to YOD identification, diagnosis, treatment, and ongoing care. The findings of this study are not only applicable to the delivery of support and care services for people with YOD and their support persons, but also to inform the design of educational resources for health professionals who are not experts in YOD.

## Background

Young onset dementia (YOD), in which dementia symptoms develop prior to 65 years of age, accounts for up to 8% of all dementia diagnoses [1]. Young onset dementia is associated with significant challenges for both the individual and their support persons, which may differ to those experienced in late onset dementia (LOD) [2]. At the time of diagnosis people with YOD are often employed, and they and their supporters may be forced to leave the workforce. This presents financial ramifications [2, 3]. They may be balancing caring responsibilities for young children and/or ageing parents and experience a shift in identity from that of a provider to a recipient of support [2, 4]. People with YOD are also often physically healthy and active yet may experience a loss of empowerment and independence as their symptoms progress [4]. Other psychological impacts include shock or embarrassment about being diagnosed with a condition commonly associated with older adults, loss of purpose, and relationship strain [4–6]. Supporters of people with YOD report greater difficulty managing dementia-related behavioural disturbances than those providing support for someone with LOD [7]. Other effects reported by support persons include stress, depression, frustration, grief, guilt, loneliness, fear of the future, and social isolation [6, 8].

People with YOD have unique needs and experiences, and as such require care to be provided by health professionals with sufficient knowledge and skills. A lack of awareness among health professionals about YOD may contribute to the average 4.7 year diagnosis delay from the onset of symptoms, with misdiagnosis being one reason [9, 10]. However recent work by O’Malley et al. has identified, using expert consensus, key elements in the diagnostic workup of YOD to aide decision making for clinicians [11]. After diagnosis, the use of formal support services in the community may delay the need for permanent residential care and can provide respite and access to peer support [3]. However, services providing dementia-related support are often designed for older adults. These services are known to lack acceptability for people diagnosed with YOD. Reasons for this include a lack of age-appropriate services, poor service accessibility (e.g. lack of transport, held during work time, lack of child care), inadequate security for physically agile participants, affordability issues, a lack of continuity of care and inadequate information provision about YOD and the support available [3, 12, 13].

It is therefore important that health professionals possess adequate knowledge and skills about YOD presentation, identification, diagnosis, treatment, and care to provide services and information that are timely, appropriate, and accessible. A preliminary step to addressing this is to determine the information that health professionals require to provide this care. This study aims to establish a consensus among those with YOD expertise about the key information that is important for health professionals to know and understand about YOD. This data can then be utilised to inform the upskilling of YOD professionals and in the development of tools to track their knowledge.

## Methods

### Design

The Delphi technique is a multistage method used to obtain a consensus among a panel of experts on a particular topic [14]. The process involves the iterative distribution of a series of questionnaires asking the participant to rank a list of items or statements in order of importance. When completing the second and subsequent questionnaires, participants are provided with the results of the previous questionnaire and are encouraged to reconsider their individual responses. The process continues until a consensus has been reached, or no further changes are being made [15, 16]. The number of iterations or “rounds” varies, but three rounds can offer a balance between rigour and participant burden [15]. The questionnaires are completed anonymously and via mail or email, minimising the risk of individual participants influencing the rest of the group [17].

In this Delphi study, three feedback rounds were conducted using an online secure survey platform. The three phases in this study were: (a) identifying key information about young onset dementia, (b) rating agreed knowledge statements, and (c) confirming group consensus via item ratings. The procedure for this study was modelled on a previous study conducted by members of our research team that identified consensus opinion about key information about dementia more generally [18].

### Participants

Participants selected for a Delphi study directly influence the quality of the data generated [15, 19]. It is vital that participants possess in-depth knowledge or experience about the topic being explored. The ideal panel size has not been established, but 10 to 30 participants is recommended in the literature [19, 20]. In this study, experts on young onset dementia were identified through networks of the research team in the areas of clinical care, psychology, research and education, advocacy and lived experience. Participants were required to have either lived experience or professional experience regarding YOD, be able to read and write in English and provide informed consent. These experts were invited to participate via an email invitation from the study team. Passive snowballing recruitment was also used meaning the experts were encouraged to forward the email invitation to others they considered experts in the area of young onset dementia who would be willing to participate in the study. The research was reviewed and approved by the Flinders University Social and Behavioural Research Ethics Committee (8331).

Experts were contacted via email from the study team throughout the project, and two follow up emails were sent for the second and third rounds. If the experts did not respond or participate in that round they were considered to have withdrawn from the study. All study participants were anonymous to each other and were identifiable to the study team only through their email address (to enable contact of participating experts at each round of the Delphi). Data collection was undertaken using an online survey platform, Qualtrics.

#### Round one (June 2020): gathering information

In the first round, the panel of experts were presented with the following open-ended questions:

- What key facts are essential to understanding young onset dementia? Participants were provided with five concept areas to consider: (a) causes and characteristics, (b) symptoms and progression, (c) assessment and diagnosis, (d) prevention and treatment, and (e) care.
- What key facts about young onset dementia are different to late onset dementia and the same as late onset dementia?
- What key facts about young onset dementia are frequently misunderstood by health professionals?

These questions were modelled on those used in the development of the Dementia Knowledge Assessment Scale (DKAS) and in consultation with the research team. The questions were selected to identify information that the experts considered important for health professionals with differing levels of knowledge and experience in YOD to understand. The responses were then independently reviewed by two researchers on the project team who collated them to produce a list of statements that reflected the range of information provided. Where possible, the experts’ own words were used to maintain authenticity and reduce researcher bias. This process resulted in a list of 48 statements representing the information that the experts deemed to be essential in understanding YOD. Statements from the Dementia Knowledge Assessment Scale [18] were also included to build on existing work.

#### Round two (August 2020): rating knowledge statements

In the second round, the statements identified in round 1 were presented to the panel of experts. They were asked to rate each statement in terms of how essential it was for knowledge of YOD among health professionals from 1 (not important at all) to 5 (very important), or N/A, indicating that they perceived that the statement was not applicable to YOD. This rating scale was selected in accordance with that used in the development of the Dementia Knowledge Assessment Scale [21]. The responses were then analysed by two researchers on the project team to calculate the level of consensus achieved for each statement.

#### Round three (November 2020): obtaining consensus

In round 3, participants were presented with the same list of statements, accompanied by each statement’s median rating (group score) and consensus level from the previous round. Participants were not informed which statements had been deemed not applicable as this was collected for analytical purposes only. Participants were asked to review this new information, and again rate each statement on the same scale of 1 to 5. The responses were then analysed to ascertain the level of consensus reached by the participants for each statement. This allowed for comparisons to be made between the results of rounds 2 and 3, where little change would indicate a stability of the consensus levels.

### Measurement and analysis

When using the Delphi technique, consensus is typically considered to have been reached when a certain percentage of the responses fall within a pre-determined range, for example 70% of participants rating 3 or higher on a four-point Likert scale [15]. The statistics commonly used in Delphi studies are measures of central tendency (mean, median and mode) and dispersion (standard deviation and interquartile range (IQR)). This allows for the combined group response to be presented, reflecting the responses of every panel member [15, 22].

This study utilised the scoring systems reported by Annear and colleagues and van der Steen and colleagues [21, 23] to build on existing work and allow for comparisons between the YOD information identified as essential in this study and that identified as essential to dementia more broadly in previous work. The scoring system is based on the median score and IQR for each statement, and the percentage of participants who scored the statement as either important or very important (the two highest levels). Full consensus was defined as a median score of 5, an IQR of 0, and 100% of participants rating the statement with the highest possible score of 5. Very high consensus was considered to be a median score of 5, an IQR of 0 and ≥ 80% scoring a 4 or 5. High consensus was defined as a median score of 5, an IQR ≤1 and ≥ 80% scoring a 4 or 5. Moderate consensus was considered to be a median score of 4–5, an IQR ≤2 and ≥ 60% scoring a 4 or 5. No consensus (low agreement) was defined as a median score of 4–5 and either IQR ≤2 or ≥ 60% scoring a 4 or 5. Statements with median scores between 2 and 4 were deemed to demonstrate no consensus (no agreement) [21, 23].

## Results

Forty-six experts on YOD were identified via professional networks and invited to participate in the Delphi study. Of those, twenty-eight individuals completed the first round (61% response rate). Sixteen of the twenty-eight participants remained in the study until completion (57% completion rate), with one additional participant completing round 3 only. Most round 1 participants were from Australia (*n* = 15, 54%), followed by Canada, the United Kingdom, the Netherlands, and Norway. Fifty-seven percent (*n* = 16) of respondents in round 1 identified themselves as being a current or retired clinician, or having dual clinical and academic roles. The clinical roles included neurologist, psychiatrist and neuropsychiatrist, psychologist, neuropsychologist and geropsychologist, physician, social worker and nurse practitioner. The remaining respondents had backgrounds in academia, advocacy, or other areas such as law, administration, homecare or were unemployed. The characteristics of the participants are summarised in Table 1.

**Table 1:** Characteristics of participants in the Delphi study

	Round 1 (***n*** = 28)	Round 2 (n = 19)	Round 3 (***n*** = 17)
***Country***			
Australia	15 (54%)	11 (58%)	10 (59%)
Canada	7 (25%)	4 (21%)	3 (18%)
Netherlands	2 (7%)	1 (5%)	1 (6%)
Norway	1 (4%)	1 (5%)	1 (6%)
United Kingdom	3 (11%)	2 (11%)	1 (6%)
Unknown	–	–	1 (6%)
***Occupation***			
Academic	2 (7%)	1 (5%)	1 (6%)
Advocate	1 (4%)	1 (5%)	1 (6%)
Other	9 (32%)	6 (32%)	4 (24%)
Unknown	–	–	1 (6%)
Clinician (current, retired or academic/clinician)	16 (57%)	11 (58%)	10 (59%)
*Neurologist*	*1 (6%)*	*1 (9%)*	*1 (10%)*
*Psychiatrist*	*1 (6%)*	*1 (9%)*	*1 (10%)*
*Consultant Neuropsychiatrist*	*1 (6%)*	*–*	*–*
*Physician*	*1 (6%)*	*1 (9%)*	*1 (10%)*
*Elderly Care Physician*	*1 (6%)*	*–*	
*Psychologist*	*4 (25%)*	*4 (36%)*	*3 (30%)*
*Clinical Neuropsychologist*	*2 (13%)*	*1 (9%)*	*1 (10%)*
*Clinical Geropsychologist*	*1 (6%)*	*–*	*–*
*Social Worker*	*1 (6%)*	*–*	*–*
*Nurse practitioner*	*1 (6%)*	*1 (9%)*	*1 (10%)*
*Not specified*	*2 (13%)*	*2 (18%)*	*2 (20%)*
***Area of expertise***^**a**^			
Research	14 (50%)	11 (58%)	10 (59%)
Clinical care	15 (54%)	11 (58%)	10 (59%)
Neuropsychology	5 (18%)	3 (16%)	3 (18%)
NDIS	2 (7%)	2 (11%)	2 (12%)
Service delivery	11 (39%)	9 (47%)	8 (47%)
Education and teaching	13 (46%)	8 (42%)	6 (35%)
Advocacy	15 (54%)	11 (58%)	8 (47%)
Lived experience	3 (11%)	2 (11%)	2 (12%)
Other	2 (7%)	2 (11%)	2 (12%)
Unknown	–	–	1 (6%)

*Abbreviations: NDIS* National Disability Insurance Scheme

^a^Participants could select multiple areas of expertise

### Round 1: gathering information

Twenty-eight participants responded to the open-ended questions presented in round 1. Their responses were independently reviewed by two researchers and collated into a list of 48 statements summarising the information provided (Table 2). These statements were then compared to those in theDKAS [18]. The DKAS was selected as it is a validated assessment of dementia knowledge based upon dementia information identified as essential using expert consensus, although not YOD-specific. Ten DKAS statements were identified as not corresponding to any of the statements based on the participant responses in this study. To utilise and build on the existing knowledge, 10 statements closely based on statements from the DKAS were added to the original 48 statements. The statements were grouped into six categories: characteristics, causes and prevention, symptoms, diagnosis, treatment, and care.

**Table 2:** Results of Round 1 - expert statements regarding essential facts to understanding young onset dementia

#	Statements
***Characteristics***	
1	Young onset dementia refers to people whose symptoms emerge prior to 65 years of age
2	Young onset dementia is not a mental illness
3	Young onset dementia accounts for 5–10% of all dementias
4	Dementias that occur secondarily to another condition (e.g. Down syndrome, heavy alcohol use) are more common in younger people than in older people
5	Young onset dementia results from physical changes in the brain
6	Mixed types of dementia are less common in younger people than in older people
7	Brain changes associated with young onset dementia usually develop several years before symptoms emerge
8	The aetiological profile of young onset dementia is more varied than for late onset dementia
9	Young people with dementia are more likely than older people to have a non-amnestic presentation (i.e. their first symptoms are less often memory-related)
10	People with intellectual disability are at high risk for young onset dementia
11	Young onset dementia is not a normal part of the ageing process^a^
12	Most forms of young onset dementia shorten a person’s life
13	Alzheimer’s disease is the most common form of young onset dementia
***Causes and prevention***	
14	Directly inherited dementias are more common among younger people than older people
15	Most cases of young onset dementia are not directly inherited
16	In most cases, young onset dementia is caused by a mix of genetic and non-genetic factors
17	Having high blood pressure increases a person’s risk of developing dementia
18	Maintaining a healthy lifestyle reduces the risk of developing the most common forms of dementia
***Symptoms***	
19	The symptoms and progression of young onset dementia will vary from person to person
20	The sudden onset of cognitive problems is NOT characteristic of common forms of dementia
21	People with young onset dementia often experience difficulty carrying out familiar home, work or leisure tasks
22	Sensory symptoms are common in young onset dementia
23	Young onset dementia causes disability
24	Movement is often affected in the later stages of young onset dementia^a^
25	People with young onset dementia may have difficulty speaking^a^
26	A person with young onset dementia may have difficulty learning new skills^a^
27	Difficulty making decisions can be a symptom of dementia
***Diagnosis***	
28	Reversible causes of impairment should be ruled out before diagnosing young onset dementia
29	People with young onset dementia are commonly misdiagnosed
30	There are no specific diagnostic markers for young onset dementia
31	Behavioural and psychological changes are key diagnostic factors for young onset dementia
32	Early diagnosis of dementia generally improves quality of life for people experiencing the condition
33	Diagnosis of dementia should include a comprehensive specialist, multi-disciplinary assessment
34	Neuropsychological testing can help to diagnose young onset dementia
35	The symptoms of young onset dementia can look like depression or another mental illness
***Treatment***	
36	There is no cure for most types of young onset dementia
37	People with young onset dementia benefit from support to remain actively engaged in their community
38	There are medications that can slow down the progression of some types of young onset dementia
39	Non-pharmacological (i.e. non-drug) treatments can help people with young onset dementia maintain their independence
40	People with YOD need tailored, specialised, multidisciplinary services to support them after diagnosis
41	Social engagement and physical activity are effective treatments for dementia
42	Non-pharmacological interventions are often the most appropriate way of treating behavioural symptoms of young onset dementia
***Care***	
43	The financial impact of having young onset dementia is significant
44	Care for people with young onset dementia should be person-centered
45	Young people with dementia require age appropriate care programs and accommodation options
46	A family approach to care is needed because many people with young onset dementia have young children in their care
47	Planning for end of life care is recommended following a diagnosis of dementia^a^
48	People with young onset dementia and their families experience more burden and negative impact of their illness than older people with dementia
49	Psychological adjustment to the diagnosis is often more difficult for young people with dementia
50	Neuropsychiatric (i.e. behavioural and psychological) symptoms are more common in young people with dementia than older people
51	Young people with dementia are more likely than older people to have a type of dementia in which neuropsychiatric (i.e. behavioural and psychological) symptoms are common
52	Uncharacteristic behaviours in a person experiencing young onset dementia are generally a response to unmet needs
53	Care partners (i.e. family and friends) of people with young onset dementia are at high risk for burden and stress
54	It is possible to communicate with a person who has advanced young onset dementia^a^
55	It is not necessary or helpful to correct a person with young onset dementia when they are confused^a^
56	People with advanced young onset dementia often communicate through body language^a^
57	Daily care for a person with advanced young onset dementia is most effective when it focuses on providing comfort^a^
58	A person experiencing advanced young onset dementia will respond to changes in their physical environment^a^

^a^ Item added from DKAS

### Round 2: rating knowledge statements

In round 2, the participants were provided with the 58 statements developed in round 1 and asked to rate the importance of each statement on a scale from 1 (not important at all) to 5 (very important), or alternatively identify the statement as being not applicable to YOD. Nineteen participants provided responses in round 2. No statements achieved full consensus, however 16 statements (28%) achieved very high consensus. Very high consensus items most commonly related to post-diagnosis care for people with YOD (*n* = 6, 38%).

### Round 3: obtaining consensus

In round 3, participants were asked to rate the same statements for a second time. They were also provided with the median rankings and consensus levels from round two. Seventeen participants completed round 3 of the study, with consensus ratings displayed in Table 3. As with round 2, no statements achieved full consensus, however all statements met some level of agreement. In total, 28% (*n* = 16) of statements achieved very high consensus, 46% (*n* = 26) reached high consensus, and 24% (*n* = 14) reached moderate consensus. Three percent (*n* = 2) of statements failed to achieve a consensus, reaching only low levels of agreement.

**Table 3:** Delphi consensus statements

Statement	Category	Median (IQR)	% Participants scoring 4/5 or 5/5 in Round 3
***Very high consensus (n = 16)*** *(median = 5, IQR = 0, ≥80% scoring 4/5 or 5/5)*			
Young onset dementia is not a normal part of the ageing process	Characteristics	5 (0)	88%
Young onset dementia refers to people whose symptoms emerge prior to 65 years of age	Characteristics	5 (0)	88%
The symptoms and progression of young onset dementia will vary from person to person	Symptoms	5 (0)	100%
Diagnosis of dementia should include a comprehensive specialist, multi-disciplinary assessment	Diagnosis	5 (0)	100%
Reversible causes of impairment should be ruled out before diagnosing young onset dementia	Diagnosis	5 (0)	94%
Neuropsychological testing can help to diagnose young onset dementia	Diagnosis	5 (0)	94%
People with YOD need tailored, specialised, multidisciplinary services to support them after diagnosis	Treatment	5 (0)	100%
People with young onset dementia benefit from support to remain actively engaged in their community	Treatment	5 (0)	100%
Non-pharmacological interventions are often the most appropriate way of treating behavioural symptoms of young onset dementia	Treatment	5 (0)	94%
Social engagement and physical activity are effective treatments for dementia^a^	Treatment	5 (0)	94%
There is no cure for most types of young onset dementia	Treatment	5 (0)	88%
Young people with dementia require age appropriate care programs and accommodation options	Care	5 (0)	100%
Care for people with young onset dementia should be person-centered	Care	5 (0)	100%
Care partners (i.e. family and friends) of people with young onset dementia are at high risk for burden and stress	Care	5 (0)	100%
A family approach to care is needed because many people with young onset dementia have young children in their care	Care		100%
The financial impact of having young onset dementia is significant	Care	5 (0)	94%
***High consensus (n = 26)*** *(median = 5, IQR ≤ 1, ≥80% scoring 4/5 or 5/5)*			
The aetiological profile of young onset dementia is more varied than for late onset dementia	Characteristics		100%
Young onset dementia is not a mental illness	Characteristics	5 (1)	94%
Young people with dementia are more likely than older people to have a non-amnestic presentation (i.e. their first symptoms are less often memory-related)	Characteristics	5 (1)	94%
Young onset dementia results from physical changes in the brain	Characteristics	5 (1)	88%
Brain changes associated with young onset dementia usually develop several years before symptoms emerge	Characteristics	5 (1)	88%
Most forms of young onset dementia shorten a person’s life	Characteristics	5 (1)	88%
People with intellectual disability are at high risk for young onset dementia	Characteristics	5 (1)	82%
Maintaining a healthy lifestyle reduces the risk of developing the most common forms of dementia^a^	Causes and prevention	5 (1)	100%
Most cases of young onset dementia are not directly inherited	Causes and prevention	5 (1)	94%
In most cases, young onset dementia is caused by a mix of genetic and non-genetic factors	Causes and prevention	5 (1)	88%
Directly inherited dementias are more common among younger people than older people	Causes and prevention	5 (1)	82%
People with young onset dementia often experience difficulty carrying out familiar home, work or leisure tasks	Symptoms	5 (1)	94%
Young onset dementia causes disability	Symptoms	5 (1)	88%
People with young onset dementia may have difficulty speaking	Symptoms	5 (1)	88%
The symptoms of young onset dementia can look like depression or another mental illness	Diagnosis	5 (1)	100%
People with young onset dementia are commonly misdiagnosed	Diagnosis	5 (1)	94%
Early diagnosis of dementia generally improves quality of life for people experiencing the condition	Diagnosis	5 (1)	88%
There are no specific diagnostic markers for young onset dementia^a^	Diagnosis	5 (1)	81%
Non-pharmacological (i.e. non-drug) treatments can help people with young onset dementia maintain their independence	Treatment	5 (1)	88%
People with young onset dementia and their families experience more burden and negative impact of their illness than older people with dementia^a^	Care	5 (1)	100%
Psychological adjustment to the diagnosis is often more difficult for young people with dementia	Care	5 (1)	94%
Uncharacteristic behaviours in a person experiencing young onset dementia are generally a response to unmet needs	Care	5 (1)	94%
Planning for end of life care is recommended following a diagnosis of dementia	Care	5 (1)	88%
It is possible to communicate with a person who has advanced young onset dementia	Care	5 (1)	82%
A person experiencing advanced young onset dementia will respond to changes in their physical environment	Care	5 (1)	82%
Young people with dementia are more likely than older people to have a type of dementia in which neuropsychiatric (i.e. behavioural and psychological) symptoms are common	Care	5 (1)	82%
***Moderate consensus (n = 14)*** *(median = 4–5, IQR ≤ 2, ≥60% scoring 4/5 or 5/5)*			
Young onset dementia accounts for 5–10% of all dementias	Characteristics	4 (1)	76%
Dementias that occur secondarily to another condition (e.g. Down syndrome, heavy alcohol use) are more common in younger people than in older people	Characteristics	4 (1)	76%
Having high blood pressure increases a person’s risk of developing dementia	Causes and prevention	4 (1)	76%
Difficulty making decisions can be a symptom of dementia	Symptoms	4 (1)	82%
The sudden onset of cognitive problems is NOT characteristic of common forms of dementia	Symptoms	5 (1)	76%
Sensory symptoms are common in young onset dementia	Symptoms	5 (2)	69%
Movement is often affected in the later stages of young onset dementia	Symptoms	4 (2)	65%
A person with young onset dementia may have difficulty learning new skills^a^	Symptoms	4 (2)	63%
Behavioural and psychological changes are key diagnostic factors for young onset dementia	Diagnosis	4 (1)	82%
There are medications that can slow down the progression of some types of young onset dementiaa	Treatment	4 (1)	77%
Neuropsychiatric (i.e. behavioural and psychological) symptoms are more common in young people with dementia than older people^a^	Care	4 (1)	93%
It is not necessary or helpful to correct a person with young onset dementia when they are confused	Care	5 (1)	76%
People with advanced young onset dementia often communicate through body language	Care	4 (1)	76%
Daily care for a person with advanced young onset dementia is most effective when it focuses on providing comfort	Care	4 (2)	65%
***No consensus (low agreement) (n = 2)*** *(median = 4–5 and (IQR ≤ 2 or ≥ 60% scoring 4/5 or 5/5))*			
Mixed types of dementia are less common in younger people than in older people^a^	Characteristics	4 (2)	56%
Alzheimer’s disease is the most common form of young onset dementia	Characteristics	4 (2)	53%

^a^At least one participant responded that this statement was not applicable to YOD

The statement that “young people with dementia require age-appropriate care programs and accommodation options” ranked highest, with 94% of participants rating the statement with the highest possible score of 5. At least one statement from each thematic category met very high consensus, except for “causes and prevention”. Of the statements that achieved very high consensus, the most prevalent themes were treatment (*n* = 5, 31%) and care (*n* = 5, 31%). Nineteen percent (*n* = 3) related to the diagnosis of YOD, 13% (*n* = 2) focused on the characteristics of YOD and 6% (*n* = 1) referred to the symptoms of YOD.

Eight statements were identified by participants as not being applicable to YOD, most commonly “there are medications that can slow down the progression of some types of young onset dementia” (*n* = 4). Two participants considered the statement “neuropsychiatric (i.e., behavioural and psychological) symptoms are more common in young people with dementia than older people” not relevant to YOD.

The additional 10 statements that were included from the DKAS [18] all reached consensus, with 10% (n = 1) reaching very high consensus, 40% (n = 4) reaching high consensus and 50% (*n* = 5) reaching moderate consensus. Although these statements were not identified spontaneously by the experts, they were nonetheless deemed as important information for health professionals to know about YOD.

## Discussion

The panel of experts in this study reached high to very high consensus on 42 statements (out of 58) that they considered to be important for health professionals to know when providing care and services to people with YOD and their families. There was agreement across all thematic categories, indicating the importance of broad professional knowledge related to YOD identification, diagnosis, treatment, and ongoing care. These data can be used to inform efforts to upskill YOD professionals and track their knowledge and skills over time.

### Identification and aetiology

Young onset dementia is an umbrella term that refers to a broad and heterogeneous range of illnesses, and in many cases occurs secondarily to another condition (for example, Huntington’s disease or alcohol use disorders) [24]. Common symptoms are not limited to cognitive impairment; depression, language impairments, and changes in behaviour or personality are regularly reported particularly by those with frontotemporal dementias [25]. Physical symptoms can include seizures, peripheral neuropathy, visual impairments, ataxia, skin lesions, headaches and visual impairment [26]. It is important for health professionals to be aware of the symptoms and stages of YOD as the range of clinical presentations and overlap of symptoms across YOD subtypes can delay the receipt of a diagnosis and often result in a dismissal of symptoms, or misdiagnosis [4, 27].

The diversity in presentation, cause, and course of YOD was evident in many of the consensus statements, including that YOD is aetiologically diverse, refers to the emergence of dementia symptoms prior to the age of 65, is distinct from a mental illness, and is not a normal part of the ageing process. Consensus was also reached for facts that are commonly mistaken about YOD, including that most cases of YOD are not directly inherited (i.e. autosomal-dominant) and have a combination of genetic and non-genetic contributing factors. However when cases of directly-inherited occur, they tend to be associated with a younger age of onset [28]. It is notable that we did not reach consensus on the statement “Alzheimer’s disease is the most common form of young onset dementia”, despite this being supported by extensive research literature [29]. This may reflect a view among our experts that the diversity in causes of YOD is more important for health professionals to know, especially as Alzheimer’s disease does not predominate in this population to the same extent as in late life.

The panel also highlighted the importance of a healthy lifestyle for reducing the risk of the most common forms of young onset dementia [21], similar to a previous Delphi study that concluded that this knowledge is essential for dementia more broadly. Several non-genetic risk factors have been identified for YOD, including low participation in cognitive leisure activities, low educational attainment, stroke, transient ischemic attack and very heavy alcohol use [30, 31]. Other potentially modifiable risk factors for dementia include physical inactivity, smoking, hypertension, obesity, diabetes, depression and low social contact [32, 33]. Awareness of these risk factors can enable health professionals to encourage lifestyle modifications that may positively ameliorate the development and clinical course of YOD.

### Diagnosis

Delayed diagnosis and misdiagnosis of YOD are very common, with delays often related to the presence of depression or mild cognitive impairment [9]. Timely and accurate diagnosis is difficult as rarer forms of dementia and non-amnestic presentations are more common at younger ages than among older people [34]. As such, it is recommended that the diagnostic process for YOD include a formal cognitive assessment, full medical history including family history, risk assessment, physical examination including neurological examination, assessment of psychiatric, psychological and behavioural symptoms, functional assessment, neuroimaging and, where appropriate, amyloid imaging and genetic biomarkers [11, 26]. This was acknowledged by our expert panel, who agreed that it was important for health professionals to know that a comprehensive, specialist, multi-disciplinary assessment is required, and identified the benefit of neuropsychological testing and the need to rule out reversible causes of impairment prior to diagnosing YOD. Knowledge about the need to conduct a comprehensive assessment will not only aid in making a timely, accurate diagnosis, but allow for the identification of co-existing symptoms and the initiation of symptom management and support services [35]. Additionally, it will provide an opportunity for accurate prognosis and future planning.

### Treatment and care

The expert panel echoed published recommendations [3, 13] that people with YOD require tailored, specialised, multidisciplinary services to support them following diagnosis. Research recommends the provision of support that addresses the physical, mental and social needs of people with YOD [32]. Programs that reduce social isolation and provide meaningful activities and continued engagement in skills or activities performed prior to diagnosis have been well received by people experiencing YOD and their supporters [3, 4, 12, 36]. Psychosocial interventions are also recommended to manage neuropsychiatric symptoms, rather than using psychotropic medications which may be ineffective and associated with adverse effects [32]. These concepts were all agreed as essential knowledge by our panel of experts.

Most importantly, the panel agreed that health professionals should be aware that young people with dementia require age-appropriate care programs and accommodation options that take a whole-family approach. People with YOD and their support persons have expressed dissatisfaction with services that are designed for older adults. Difficulty relating to older participants, lack of security for physically agile people with YOD, and services being offered during business hours without childcare have been identified as reasons for which services targeted at people with LOD are not appropriate for people with YOD [3]. Previous research has recommended that care instead be tailored to the individual and also their family and/or support persons [4, 26]. Support persons of people with YOD report very high rates of stress and burden [6, 37] and children can experience a loss of care from both parents as a result.

Finally, our panel emphasised the significant financial impact of YOD. People with YOD may have to leave the workforce earlier than planned and before they are eligible to receive superannuation or pensions. This may result in their care partner being required to increase their working hours, or they may need to cease work to support them [5, 6, 26]. Research has shown that the impact of this can result in reduced access to services from diagnosis through to placement in residential care [3]. Holistic care for people with YOD therefore requires that health professionals are aware of these additional impacts on family members and friends.

### Strengths and limitations

This international Delphi consensus study provides expert guidance about the knowledge that professionals working with people with YOD, and their families need to have to provide best-practice care. There are nonetheless important limitations to this work. Although many countries were represented, a large proportion of participants lived in Australia. This may introduce a geographical bias, and a more equal distribution of countries may provide a greater range of responses and experiences upon which to draw. Another limitation was the predominance of researchers and clinicians in the sample, particularly by round 3. Greater representation from health professionals providing YOD care in the disability and aged care sectors and individuals with a lived experience of YOD may provide a more thorough exploration of the topic. An established limitation of the Delphi study methodology is that it can be time consuming for participants to undertake. This may have contributed to the reduction in response rate over the course of this study. However, this is an issue common to other published Delphi studies [20, 38–40] and the number of participants who completed round 3 within the recommended sample size for this method [19, 20].

## Conclusions

This international Delphi study has established the key pieces of information that experts consider essential for health professionals to understand about YOD to enable to them to deliver best-practice care. The statements indicate the breadth of knowledge that health professionals should be expected to know and can be used to guide the design and delivery of diagnostic, treatment, and support services for people with YOD and their support persons. Additionally, the statements can be used in the development of training and education materials to improve the awareness and understanding of YOD among health professionals providing care to this varied group of clients.

## Data Availability

The datasets used and/or analysed during the current study are available in a de-identified format from the corresponding author on reasonable request.
